# Optothermal Switching of Cholesteric Liquid Crystals: A Study of Azobenzene Derivatives and Laser Wavelengths

**DOI:** 10.3390/ma8095293

**Published:** 2015-09-11

**Authors:** Tai-Chieh Huang, Yen-Yu Chen, Chih-Chien Chu, Vincent K. S. Hsiao

**Affiliations:** 1 Department of Applied Materials and Optoelectronic Engineering, National Chi Nan University, Nantou 54561, Taiwan; E-Mail: s99328044@mail1.ncnu.edu.tw; 2 Graduate Program of Optoelectronic Technology, National Chi Nan University, Nantou 54561, Taiwan; E-Mail: Tanaka.Chen@auo.com; 3 Department of Medical Applied Chemistry, Chung Shan Medical University, Taichung 40201, Taiwan

**Keywords:** optothermal, azobenzene, anthraquinone dye, cholesteric liquid crystal

## Abstract

The laser-initiated thermal (optothermal) switching of cholesteric liquid crystals (CLCs) is characterized by using different azobenzene (Azo) derivatives and laser wavelengths. Under 405-nm laser irradiation, Azo-doped CLCs undergo phase transition from cholesteric to isotropic. No *cis*-to-*trans* photoisomerization occurs when the 405-nm laser irradiation is blocked because only a single laser is used. The fast response of Azo-doped CLCs under the on–off switching of the 405-nm laser occurs because of the optothermal effect of the system. The 660-nm laser, which cannot be used as irradiation to generate the *trans*–*cis* photoisomerization of Azo, is used in Anthraquinone (AQ)-Azo-doped CLCs to examine the optothermal effect of doped Azo. The results show that the LC-like Azo derivative bearing two methyl groups ortho to the Azo moiety (A4) can greatly lower the clearing temperature and generate large amount of heat in AQ-A4-doped CLCs.

## 1. Introduction

Photoresponsive liquid crystals (PLCs) [1], which consist of a photochromic dye and LCs (nematic LCs, cholesteric LCs, and blue phase LCs), have attracted the attention of researchers because several optical properties, such as light intensity, polarization, and reflective wavelength, can be modulated or switched by light-induced LC phase changes. Light, the external stimulus, can perform noncontact and small-area irradiation that is suitable for light-activated LC-based micro- or nanodevices [2,3,4,5,6,7,8,9,10,11]. For example, in a mixed nematic LC, an azobenzene (Azo)-based photochromic dye composed of two phenyl rings, UV-light irradiation produces the *cis*-Azo (a bent-shaped isomer), which changes the LC phase from nematic to isotropic [1]. However, visible-light irradiation generates the *trans*-Azo (rod-shaped isomer) and thus reverts the LC phase to nematic. The transmittance, polarization, and optical bandgap from Azo-dye-doped cholesteric LCs (CLCs) can be modulated under alternative UV and visible-light irradiation [12,13,14,15,16,17,18,19,20,21,22,23,24]. Recent studies have shown that using a single laser beam (405-nm wavelength) can generate an efficient external stimulus to modulate the optical properties from Azo-dye-doped CLCs. The fast on–off response (<1 s) from the sample could be achieved by using the alternative on–off irradiation of a single laser beam [25,26]. However, without using visible-light irradiation, thermally reverting a *cis*-Azo isomer to a *trans*-Azo isomer requires more than 1 h. Determining why a single laser beam can be used as light irradiation to generate this fast switching response can improve the performance of PLC systems. In this paper, we prove that in PLC systems using a single laser beam as irradiation, the addition of Azo derivatives decreases the clearing temperature (CT) of CLCs. Under 405-nm wavelength laser irradiation, the generation of *cis*-Azo (a bent-shaped isomer) disturbs the CLC orientation and further decreases the CT. The temperature from the laser exposure increases because of the laser-induced thermal (optothermal) effect of the Azo derivatives. The phase transition from cholesteric to isotropic is initiated by the combination of the decreasing CT and optothermal effect. When the laser is further switched off, the temperature decreases, and the CLC phase reverts from isotropic to cholesteric because of the thermal isomerization of Azo derivatives. A 660-nm wavelength laser, which cannot generate *trans*–*cis* photoisomerization resulting from Azo, was used as light irradiation to further prove the concept of optothermal switching. Anthraquinone (AQ) dye [27], which generated heat by absorbing the 660-nm wavelength laser, was added to the Azo-doped CLCs. The control experiment (without the addition of Azo) shows that no CLC phase transition was observed under 100 mW/cm^2^ laser irradiation (660 nm). The addition of Azo decreases the CT and assists in the phase transition of AQ-doped CLCs under the same intensity of laser irradiation. In combination with the previous results, which showed that a 250 mW/cm^2^ laser intensity was necessary to generate the optothermal switching of the CLCs, our results can be applied to reduce the laser intensity for the optothermal switching systems of AQ-doped LC-based devices.

## 2. Results and Discussion

**Figure 1:**
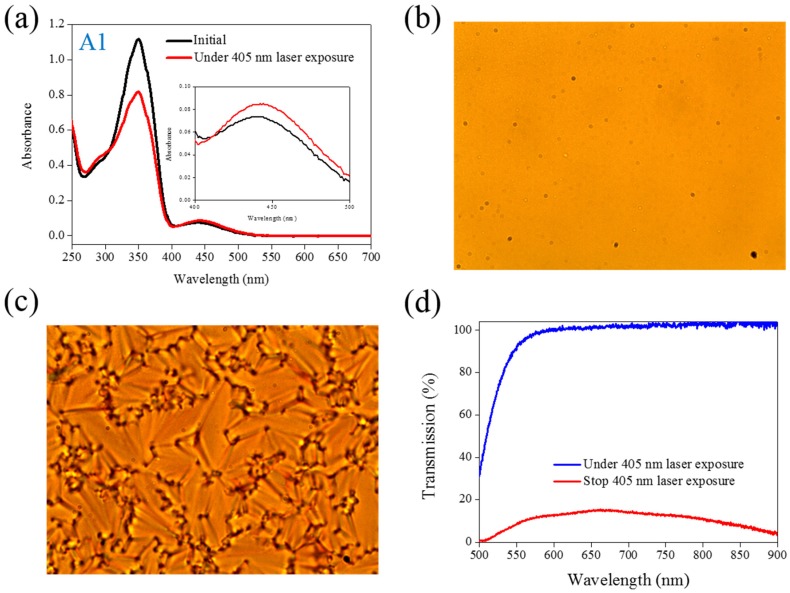
(**a**) Absorption spectral response of A1 of $1\times 10^{-5}M$ in the tetrahydrofuran (THF) solution before and under 405-nm laser irradiation; (**b**,**c**) Typical POM images (picture area: 270 μm × 320 μm) of A1-doped CLC (15 wt% A1, 20 wt% ZLI811, and 65 wt% MDA3461) under (**b**) and stop (**c**) 405-nm laser exposure; (**d**) The transmission spectra of A1-doped CLC under different conditions of laser exposure.

UV light of a 360-nm peak wavelength is typically used to initiate the photoisomerization of Azo dye because the Azo molecule exhibits a strong absorption peak of a 350-nm wavelength. Under UV light exposure, the intensity of a $\pi -\pi^{*}$ band located at a 350-nm wavelength decreases, whereas the intensity of an $\pi -\pi^{*}$ band located at a 450-nm wavelength increases. Visible light of a 450-nm peak wavelength must be used to initiate the back photoisomerization. The optical properties of the Azo dye-doped LC, such as optical polarization, could be on–off switching under alternative UV and visible light irradiation. Our previous studies have proven that the use of a 405-nm laser could also initiate the photoisomerization of Azo-LCs (A1, BMAB), as shown in Figure 1a, even though the Azo-LC has weak absorption at a 405-nm wavelength. Without the use of the second light irradiation, the optical properties of Azo-LC-doped CLCs could be on–off switching under on–off exposure of a 405-nm laser (80 mW/cm^2^). Figure 1b shows the POM image of A1-doped CLCs under 405-nm laser light exposure. The light-initiated bend-like structure of A1 (*cis*-A1) disrupts the CLCs and causes the LC cell to be transparent. When the laser light was switched off, the CLCs recovered, and a focal conic CLC phase was formed, as shown in Figure 1c. Figure 1d shows the transmittance change of A1-doped CLCs before and under 405-nm laser irradiation, and an 80% transmittance change could be achieved under on–off laser exposure.

**Figure 2:**
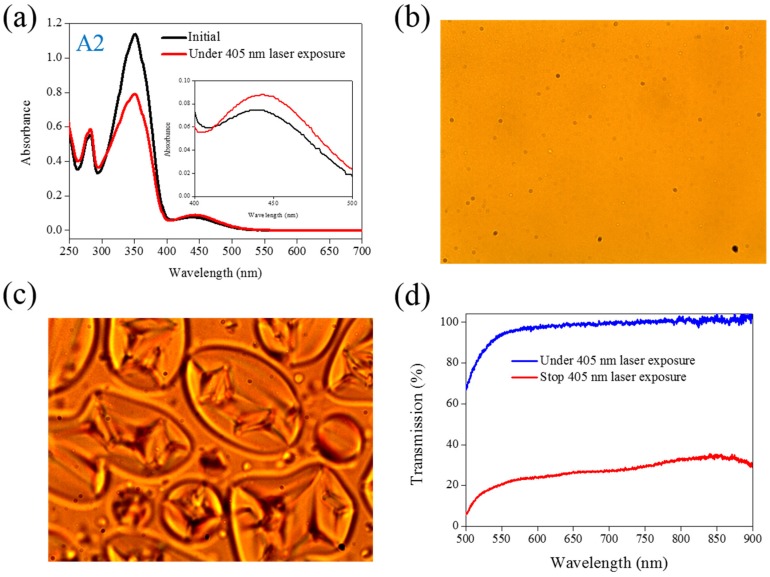
(**a**) Absorption spectral response of A2 of $1\times 10^{-5}M$ in the THF solution before and under 405-nm laser irradiation; (**b**,**c**) Typical POM images (picture area: 270 μm × 320 μm) of A2-doped CLC (15 wt% A2, 20 wt% ZLI811, and 65 wt% MDA3461) under (**b**) and stop (**c**) 405-nm laser exposure; (**d**) The transmission spectra of A2-doped CLC under different conditions of laser exposure.

**Figure 3:**
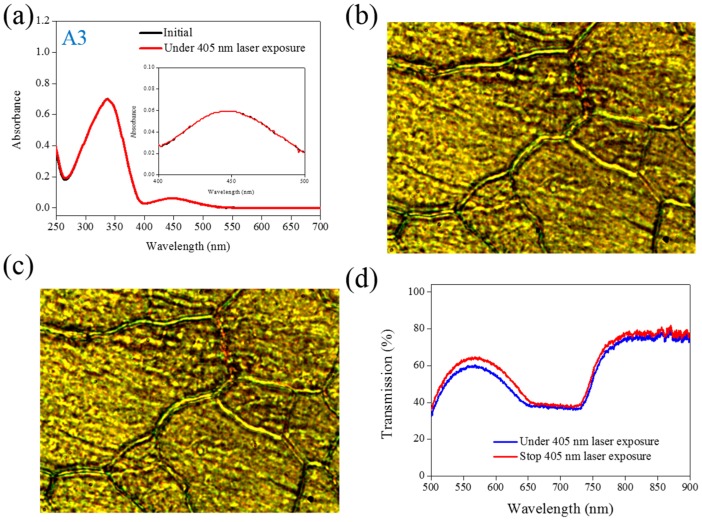
(**a**) Absorption spectral response of A3 of $1\times 10^{-5}M$ in the THF solution before and under 405-nm laser irradiation; (**b**,**c**) Typical POM images (picture area: 270 μm × 320 μm) of A3-doped CLC (15 wt% A3, 20 wt% ZLI811, and 65 wt% MDA3461) under (**b**) and stop (**c**) 405-nm laser exposure; (**d**) The transmission spectra of A3-doped CLC under different conditions of laser exposure.

**Figure 4:**
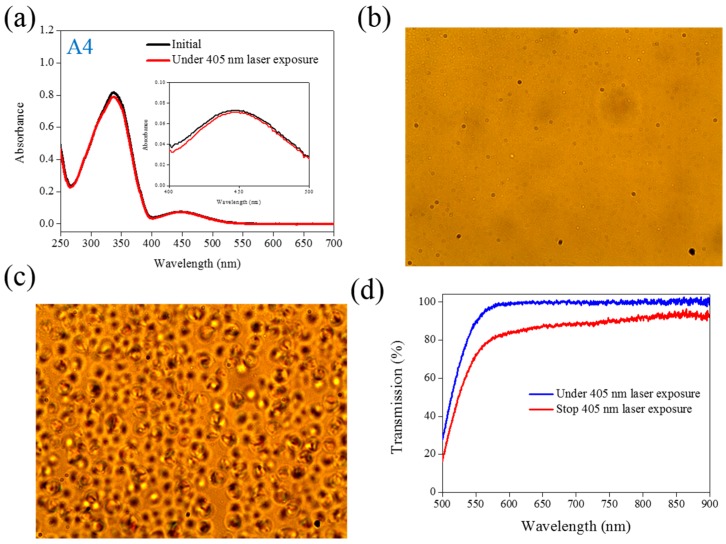
(**a**) Absorption spectral response of A4 of $1\times 10^{-5}M$ in the THF solution before and under 405-nm laser irradiation; (**b**,**c**) Typical POM images (picture area: 270 μm × 320 μm) of A4-doped CLC (15 wt% A4, 20 wt% ZLI811, and 65 wt% MDA3461) under (**b**) and stop (**c**) 405-nm laser exposure; (**d**) The transmission spectra of A4-doped CLC under different conditions of laser exposure.

The same experiments have been performed in A2, A3, and A4 compounds. The use of A2 involves behavior similar to that of A1, as shown in Figure 2. Under 405-nm laser irradiation, the generated *cis*-A2 changes the LC phase from cholesteric to isotropic. Unlike A1 with LC like, linear shape molecular structure, the ability of generating *cis*-form Azo from A2 (bulky and dentrilic structure) is lower than A1. The use of A3 involves behavior different from that involved in the use of A1 and A2. The absorption spectrum of A3 remains the same before and under 405-nm laser irradiation, as shown in Figure 3a. The lack of C_4_H_9_ chain prohibits the occurrence of *trans*–*cis* photoisomerization from A3 compound. Because the *cis*-form of A3 was not generated under 405-nm laser irradiation, the CLC phase of planner texture in the A3-doped CLC sample remains unchanged, as shown in Figure 3b,c. No focal conic texture was formed in A3-doped CLCs under 405-nm laser irradiation. The transmission spectrum of the sample changed slightly under 405-nm laser exposure, as shown in Figure 3d. Figure 4 shows the experimental results from the A4-doped CLC sample. Under 405-nm laser irradiation, the $\pi -\pi^{*}$ band slightly decreases, whereas the $\pi -\pi^{*}$ also slightly decreases. Compared with the effects resulting from the use of A1 and A2, the effect of photoisomerization under 405-nm laser exposure was very weak in the A4-doped CLC sample. However, a CLC phase of focal conic texture was formed in the A4-doped CLC sample after 405-nm laser exposure, as shown in Figure 4c. Because the 405-nm laser could not initiate the *trans*–*cis* photoisomerization of the A4 compound, the phase change between the focal conic and isotropic phases may be caused by the optothermal effect that the effect was verified by measuring the sample’s temperature. The A4-doped CLC sample showed the highest temperature (38.9 °C) under 405-nm laser exposure.

**Figure 5:**
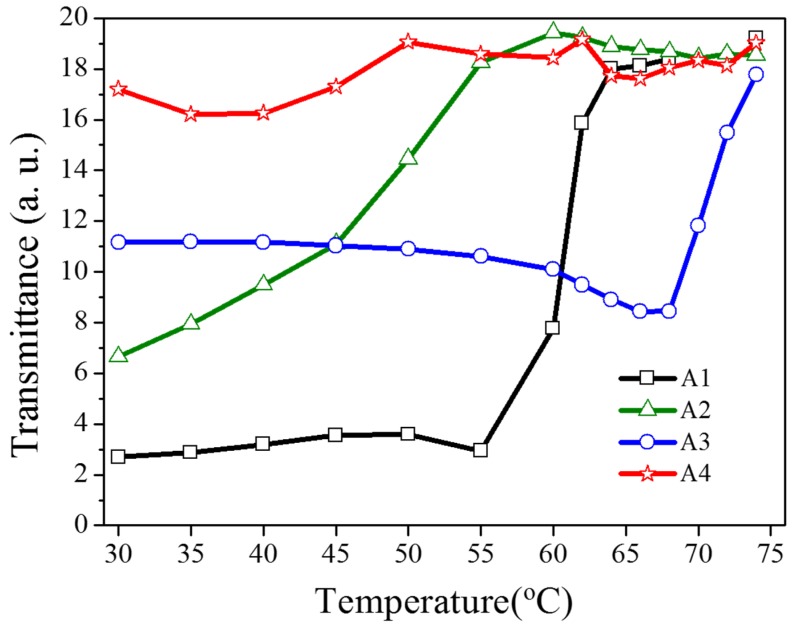
Transmittance of Azo-doped CLC samples dependent on the heating temperature. The order of CT-lowering ability is A4 > A2 > A1 > A3.

**Figure 6:**
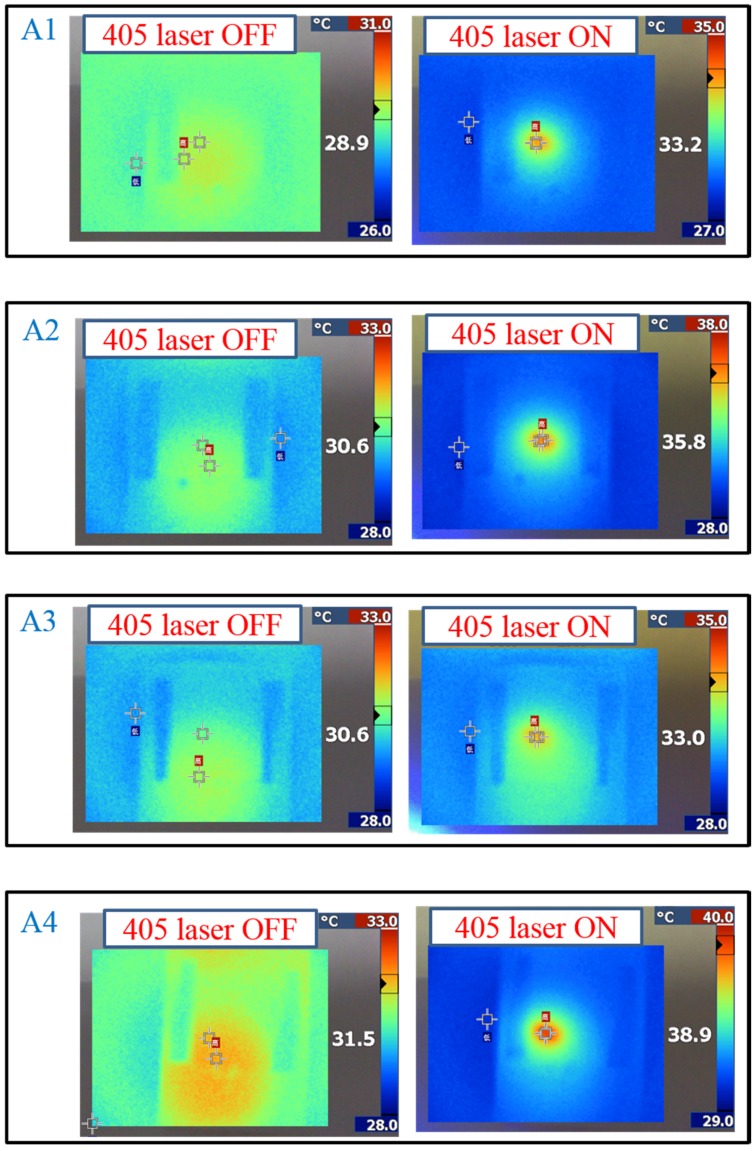
Temperature change of Azo-doped CLC samples under different conditions of laser exposure. The order of temperature change is A4 > A2 > A1 > A3.

As shown in Figure 5, the transmittance dependent on the heating temperature was recorded to verify the optothermal effect from the Azo-doped CLCs used in this study. For example, regarding A1, before the temperature of the heating plate reaches 55 °C, the transmittance of the sample remains unchanged, indicating that the CT is higher than 55 °C. The A3 compound has a higher CT than A1 does because the transmittance increases when the temperature of the heating plate reaches 68 °C. When the CT is defined as the temperature required to cause the entire sample to be transparent, the CT-lowering ability is A4 (50 °C) > A2 (60 °C) > A1 (65 °C) > A3 (75 °C), where A4 has the lowest CT, which induces a stronger optothermal effect. Figure 6 shows the sample temperature recorded by the thermal imager (Fluke Ti30) before and under 405-nm laser exposure, and the A4-doped CLCs show that the highest temperature was 38.9 °C under laser exposure. The order of temperature induced by the Azo compound is A4 (7.4 °C) > A2 (5.2 °C) > A1 (4.3 °C) > A3 (2.4 °C). The photo-induced tuning mechanism of using single laser (405 nm) to carry out the switching effect contains two processes. Under 405 nm laser exposure, the *trans*-to-*cis* photoisomerization occurs and the *cis*-form Azo disrupts the CLC and change the LC phase from cholesteric to isotropic. Once the *cis*-form of Azo is generated, back *cis*-to-*trans* photoisomerization occurs only if another laser of longer wavelength is used. Because 405-nm wavelength laser cannot efficiently trigger the photoisomerization comparing to UV light, which is located in the peak absorption of Azo, most of the laser energy converts to thermal energy. The temperature of sample increases under laser exposure, as shown in Figure 6. Turning off 405-nm laser irradiation changes the LC phase from isotropic to focal conic texture due to the back thermal isomerization.

**Figure 7:**
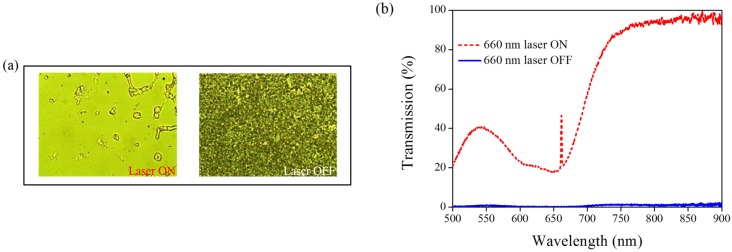
(**a**) POM images (picture area: 270 μm × 320 μm) and (**b**) transmission spectra of AQ dye-doped CLC containing A4 molecules (3 wt% AQ dye, 15 wt% A4, 20 wt% S811 and 62 wt% MDA3461) under different conditions of 660-nm wavelength laser exposure. The laser intensity was 100 mW/cm^2^.

**Figure 8:**
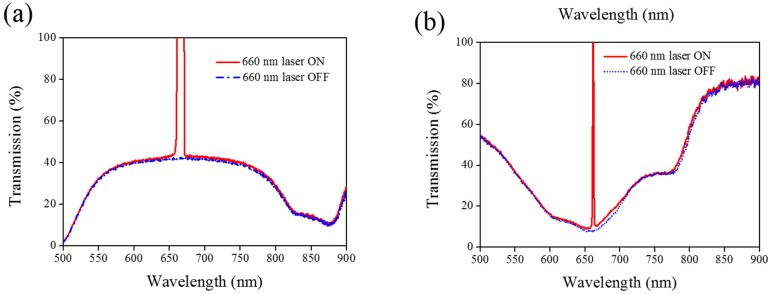
Transmission spectra of the sample without the addition of (**a**) AQ dye and (**b**) A4 under different conditions of laser exposure.

**Figure 9:**
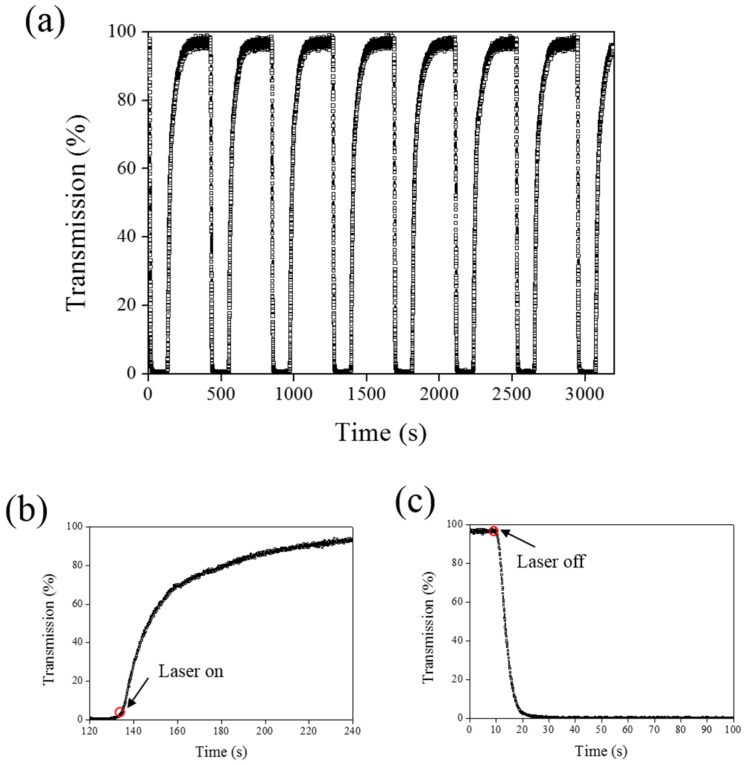
(**a**) Switching performance of AQ-A4-doped CLC under alternative on–off laser irradiation (660 nm); (**b**) time-dependent transmittance change under laser exposure; and (**c**) time-dependent change as turning off the laser light.

A 660-nm laser diode of 100 mW/cm^2^ intensity and AQ dye were used to further verify the optothermal ability of A4 molecules in Azo-doped CLCs. AQ dye has been used to generate optothermal effects in AQ dye-doped CLCs under 660-nm laser exposure [27]. However, a large Kr^+^ laser (647 nm) and high intensity of 250 mW/cm^2^ were used to initiate the switching effect that limits the application of optothermal systems in dye-doped CLCs. Figure 7 shows the phase and transmittance change of CLCs containing both Azo (A4) and AQ dye. An isotropic and focal conic phase could be switched under on–off single 660-nm laser exposure, as shown in Figure 7a. Figure 7b shows the transmission spectrum of the sample before and under 660-nm laser exposure. Before laser exposure, a focal conic CLC texture scatters the light, and a nearly 0% transmittance was observed. Under laser exposure, the transmittance increases and reaches nearly 100% (wavelength higher than 800 nm). The transmission spectrum of the laser-on sample shows a deep notch between 550 nm and 750 nm, indicating the absorbance from the AQ dye. The control experiments were performed to verify the role of AQ and Azo dye (A4). Figure 8 shows the transmission spectra from the CLC sample, which separately contains only A4 (Figure 8a) and only AQ dye (Figure 8b). Both samples show no transmittance change under 660-nm laser exposure. The addition of AQ dye assists in the absorption of a 660-nm laser, whereas the addition of A4 decreases the CT that assists in lowering the irradiated laser intensity. Figure 9 shows the time-dependent transmittance change of the sample at 800 nm with and without laser irradiation. The transmittance could be switched between 0% and 100% at an 800-nm wavelength under on–off laser exposure, as shown in Figure 9a. The response time is separately 100 s and 10 s under on–off switching laser exposure, as shown in Figure 9b,c.

**Figure 10:**
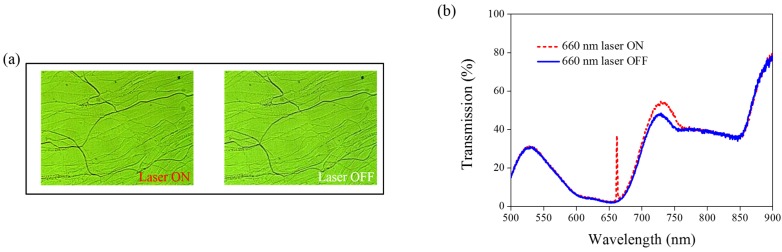
(**a**) POM images (picture area: 270 μm × 320 μm) and (**b**) transmission spectra of AQ dye-doped CLC containing A1 molecules (3 wt% AQ dye, 15 wt% A1, 20 wt% S811, and 62 wt% MDA3461) under different conditions of 660-nm wavelength laser exposure. The laser intensity was 100 mW/cm^2^.

**Figure 11:**
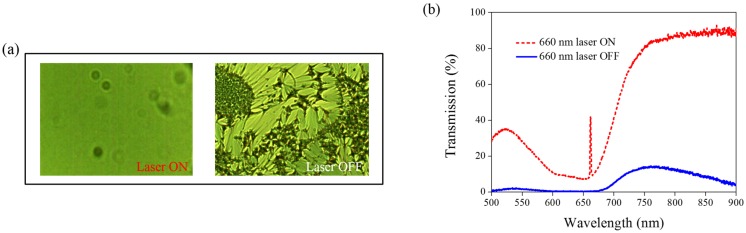
(**a**) POM images (picture area: 270 μm × 320 μm) and (**b**) transmission spectra of AQ dye-doped CLC containing A2 molecules (3 wt% AQ dye, 15 wt% A2, 20 wt% S811, and 62 wt% MDA3461) under different conditions of 660-nm wavelength laser exposure. The laser intensity was 100 mW/cm^2^.

**Figure 12:**
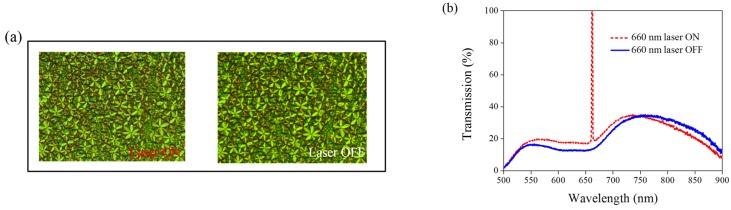
(**a**) POM images (picture area: 270 μm × 320 μm) and (**b**) transmission spectra of AQ dye-doped CLC containing A3 molecules (3 wt% AQ dye, 15 wt% A3, 20 wt% S811, and 62 wt% MDA3461) under different conditions of 660-nm wavelength laser exposure. The laser intensity was 100 mW/cm^2^.

Other Azo derivatives, A1, A2, and A3, have also been incorporated into the optothermal system of AQ-dye-doped CLCs, as shown in Figure 10, Figure 11 and Figure 12. No obvious transmittance change was observed under 660-nm laser exposure from the sample containing A1 and A3 that had a higher CT, as shown in Figure 5, and low optothermal effect, as shown in Figure 6. For the sample containing A2, 60% transmittance was observed, and the results were consistent with the observations from Figure 5 and Figure 6, which show that the CT of A2 is lower than those of A1 and A3, and the temperature change from the A2-doped sample is larger than those of the A1- and A3-doped samples.

## 3. Experimental Section

**Figure 13:**
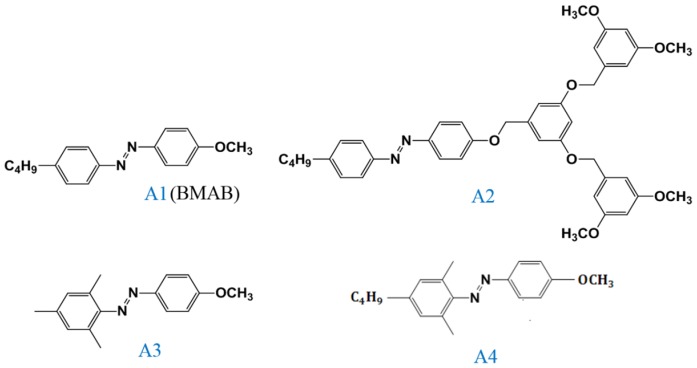
Structures of the LC-like Azo derivatives and their abbreviations.

The NLC (Merck E-series, MDA 3461, n_e_ = 1.77, n_0_ = 1.51 at 589 nm) (Merck Taiwan, Taipei, Taiwan) was used as the LC host, and the CT of MDA 3461, which is defined as the temperature when the LC phase changes from nematic to isotropic, is 92 °C. The left-handed (ZLI 811) and right-handed (S 811) chiral molecule of fixed 20 wt% were dissolved in the NLC host to produce a cholesteric phase. The LC-like Azo (A1, A2, A3, and A4) were synthesized according to the reported paper [28]. The structure of the Azo derivatives used in this study are shown in Figure 13. Transmission-mode analysis was used to measure the CT of the CLC samples. The intensity of the probe beam (5 mW, 633 nm, He-Ne laser) (Edmund Optics Taiwan Branch, Taichung, Taiwan) passing normally to the samples was monitored by a power meter while the sample was heated by a transparent hot plate. The transmission spectra were recorded using a fiber-based UV-VIS spectrometer (Ocean Optics, HR4000HCG) (Dunedin, FL, USA). The phototunable properties of the Azo-doped CLC cells with or without the AQ dye were characterized by monitoring the optical transmittance change of the CLC mixture in the light “on” and “off” states. The switching behavior of the CLC cell was characterized by recording variations of transmittance in the transmission spectra at a wavelength of 800 nm. The polarization optical microscopic (POM) images were recorded using a polarized microscope (Olympus IX 71, Olympus Taiwan Co., Ltd., Taichung, Taiwan) equipped with a CCD camera under the observation of crossed polarizers. A 405-nm wavelength laser diode (80 mW/cm^2^) was used to characterize the PLC system using only Azo dye as light absorbing material. A 660-nm wavelength laser diode (100 mW/cm^2^) was used to characterize the PLC system using AQ dye as light-absorbing material. The laser exposure time was 2 min. All PLC samples were prepared in 12-μm-thick sandwiched glass cells without orienting substrates. All measurements were performed at room temperature.

## 4. Conclusions

The optothermal system of Azo- and AQ-doped CLCs was characterized, and the results show that the use of suitable Azo helps to decrease the CT of CLCs and further facilitates the phase change under laser irradiation. For the A4-doped CLCs, no obvious phase change was observed under 405-nm laser irradiation. Because no *trans*-to-*cis* photoisomerization occurred in the sample under 405-nm laser exposure, the slight transmittance change was caused by the laser-induced thermal effect. The AQ-doped CLCs containing A4 molecules further proves the strong optothermal effect of the A4 molecules. The large temperature change and lower CT caused partially by the A4 molecules facilitates the phase change in the CLC sample containing both AQ and A4 molecules under 660-nm laser exposure. The demonstrated optothermal system can be applied in LC-based, light-switchable micro- or nano-devices.
